# A Single-System Ectopic Ureter in a Child: A Challenge for Early Diagnosis

**DOI:** 10.7759/cureus.51834

**Published:** 2024-01-08

**Authors:** Barbara A Reinig, Bianca M Silva, Marilyse Fernandes, Ana Luiza Onofre, Erika Veruska Paiva Ortolan

**Affiliations:** 1 General Surgery, Hospital das Clinicas de Botucatu, Botucatu, BRA; 2 General Surgery, Hospital das Clínicas de Botucatu, Botucatu, BRA; 3 Pediatric Surgery, Hospital das Clínicas de Botucatu, Botucatu, BRA; 4 Surgery, Hospital das Clínicas de Botucatu, Botucatu, BRA

**Keywords:** congenital anomaly of urinary tract, pediatric urology, pediatric surgery, congenital anomaly of kidney and urinary tract, urinary incontinence (ui), ureter ectopic insertion, ectopic ureter

## Abstract

An ectopic ureter is an uncommon anomaly, usually associated with a duplicated urinary system. Up to 20% of ectopic ureters occur in a single system. In females, only 25% of ectopic ureters insert into the vagina and usually cause urinary incontinence, which can be confused with vaginal discharge. The diagnostic investigation includes urinary tract ultrasound, DMSA, and urethrocystography, which evaluate renal morphology and function, determining factors for surgical treatment decision that aims to preserve renal function, prevent the recurrence of infections, and reestablish urinary continence. The rarity of this anomaly and the delay in recognizing symptoms are factors related to late diagnosis.

## Introduction

An ectopic ureter is a congenital anomaly in which the ureter inserts outside the bladder trigone. The incidence is between 1:2000 and 1:4000 of the population; 80% of cases occur in the setting of a duplicated system and are more prevalent in females in a ratio of 7:1 [1,2]. In males, the ectopic insertion of the ureter is always above the external sphincter, especially in the prostatic urethra (54%), and may insert into the seminal vesicles (28%), vas deferens (10%), or ejaculatory duct (8%). In females, ectopic insertion is usually below the sphincter, which causes urinary incontinence, with insertion sites in the upper urethra (33%), vestibule (33%), vagina (25%), and less commonly in the uterus or cervix (5%) [3].

The embryological origin of the ectopic ureter is related to the initial position of the ureteral bud in the Wolffian duct and the abnormal development of nephrogenic blastema, which occurs in the fourth week of gestation [4]. In males, due to the ectopic insertion of the ureter being above the external sphincter, it usually presents with obstructive conditions and urinary infections. In females, when the insertion of the ectopic ureter occurs in the genital tract, it usually presents with urinary incontinence or pseudo incontinence. The constant dribbling of urine interval between normal micturition can be confused with vaginal discharge, a symptom usually neglected by the patient, delaying diagnosis [1].

Physical examination is often normal, but in female patients may reveal an ectopic ureter opening into the vulva or vestibule. In pre-teens and teens, we can perform a pad test, which is the best way to establish that the incontinence is continuous [5,6]. Urinary ultrasonography is the first-line investigation and may show ureterohydronephrosis, lower implantation of the ureter, and a kidney with atrophic or dysplasic aspects. Urethrocystrography should be included in the investigation because 70% of cases may present reflux and the exam shows the anatomy of the ectopic ureter associated with the renal functioning segment. In cases where the affected segment is dysfunctional, magnetic resonance imaging plays an important role in the anatomical and functional analysis of the kidney, but anesthesia is necessary in children [1,2,5].

The primary goal in the surgical treatment of ectopic ureters is to preserve kidney function, prevent recurrent episodes of infection, and restore continence. Surgical planning will depend on the functional status of the kidney and anatomy (duplicated or single-system) and can be performed by open, laparoscopic, or endoscopic techniques. If the renal segment drained by the ectopic ureter is functional, nephron preservation techniques should be preferred, such as ureteroneocystostomy and ureteroureterostomy. If the segment is not functional, nephroureterectomy can be considered [1,4,5].

## Case presentation

We present a four-year-old female who had two episodes of febrile urinary tract infection in the first year of life, both treated under inpatient care. A urinary tract ultrasound revealed a left renal pelvis dilatation (AP 7mm), without calyceal clubbing or cortical thinning, associated with a dilated left ureter and mild left hydronephrosis. The patient was referred to the genitourinary anomalies in the Pediatric Surgery Outpatient Clinic of the "Hospital das Clínicas de Botucatu (UNESP)" at the age of three years due to suspicion of obstructive megaureter. The static renal scintigraphy (DMSA) showed a renal function in the right kidney of 55.62% and in the left kidney of 44.38%, with homogeneous uptake of radiotracer and no renal scarring. The renal dynamic scintigraphy (DTPA renogram) showed reduced uptake of radiotracer with excretion to tortuous and dilated left ureter. The micturating cystourethrogram did not reveal bladder abnormalities or the presence of vesicoureteral reflux. The doctors chose to maintain outpatient follow-up due to satisfactory renal function and the absence of new episodes of febrile urinary tract infection in the last two years. 

**Figure 1:**
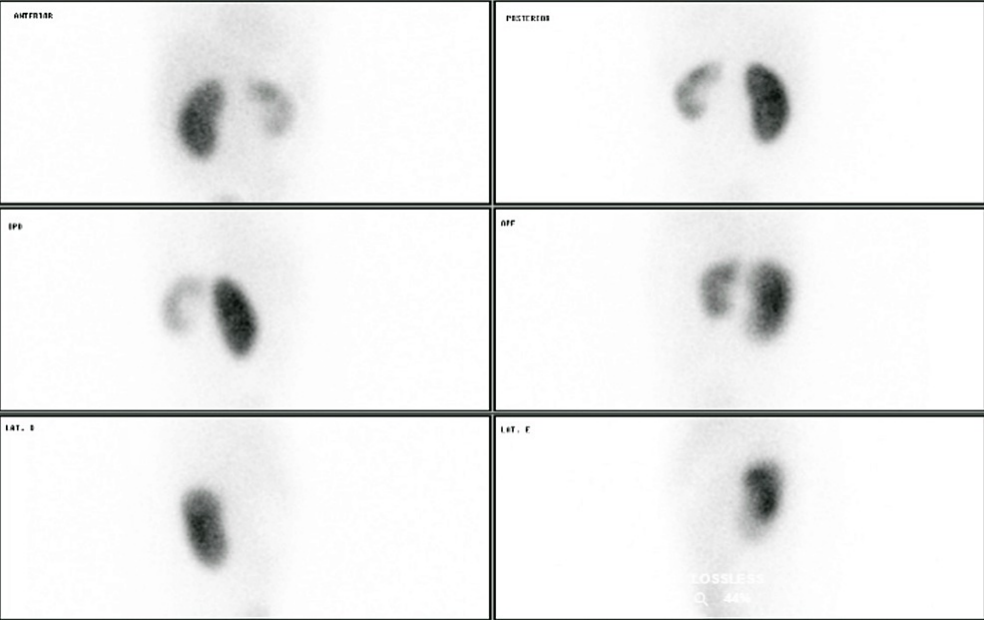
DMSA showing abnormal renal morphology and a reduced uptake of radiotracer in the left kidney in different positions. DMSA: Static renal scintigraphy

After six months of follow-up, the patient's mother reported difficulties with toilet training, due to complaints of mictional urgency and urinary incontinence. The patient was submitted to a new DMSA test, which showed an estimated quantitative relative renal function in the right kidney of 78.64% and in the left kidney of 21.36% and moderate tubular dysfunction with probable cortical scarring upper pole left kidney (Figure 1). A new urinary tract ultrasound demonstrated maintenance of the left renal ectasia pattern and an increase in the left ureter caliber, without calyxes' dilatation.

**Figure 2:**
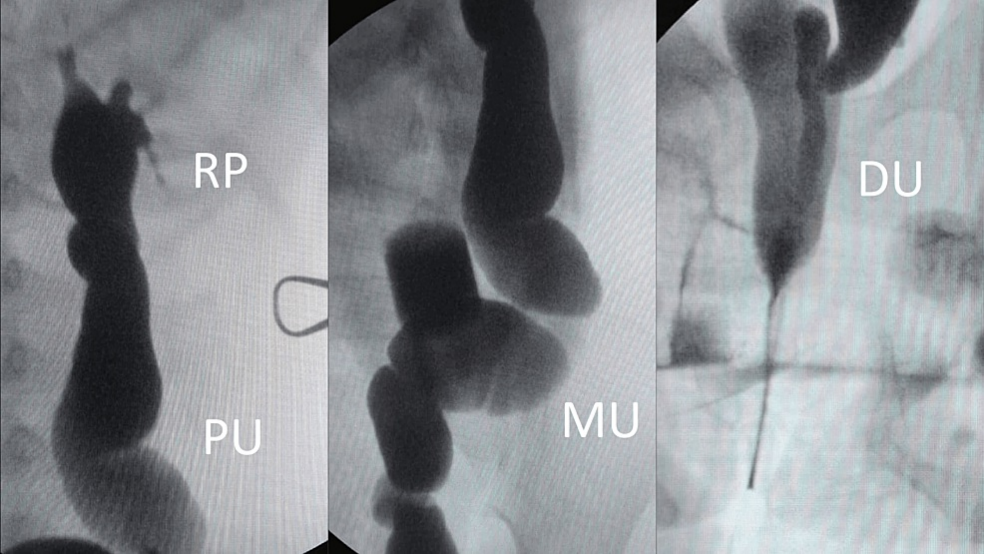
Intraoperative retrograde pyeloureterography showing dilated and tortuous left ureter with distal segment following caudally to the pelvis. RP: Renal pelvis. PU: Proximal ureter. MU: Mid ureter. DU: Distal ureter.

Given the worsening of renal function and the progression of left ureter dilatation, the pediatric surgery team decided on surgical treatment with ureteral reimplantation via an extravesical approach (Lich-Gregoir technique), faced with the hypothesis of obstructive megaureter. During the surgical procedure, the absence of an internal orifice of the ureter in the bladder was observed, then a dissection of the left ureter was performed, which went caudally to the pelvis and with no ureteral duplication (single-system). The surgical team performed an excision of the distal ureter and realized an intraoperative retrograde pyeloureterography, which showed a dolichal ectopic left ureter tortuous and enlarged in caliber (approximately 15mm) (Figure 2). The opening site was identified by the extravasation of contrast through the vaginal canal. The final surgical treatment was ureteral remodeling and reimplantation in the left posterolateral inferior bladder wall with an anti-reflux technique. The patient presented urinary continence on the first postoperative day and completed the toilet training in one week.

## Discussion

An ectopic ureter is an uncommon condition, occurring in 1:2000-4000 of the population, more prevalent in females and usually associated with a duplicated system. A single-system ectopic ureter accounts for less than 20% of cases, and this is more prevalent in males [1,2]. In women, the most common sites of ectopic insertion of the ureter are the upper portion of the urethra and vestibule, followed by vaginal insertion in 25% [3]. In this case report, a single-system ectopic ureter in a female with ureteral insertion into the vagina represents a rare occurrence.

Female patients with ureteral ectopy usually present with episodes of urinary infections, urinary incontinence, and pseudo incontinence, but they may have normal micturition with constant dribbling of urine and be mistaken by vaginal discharge, usually resulting in diagnosis in older ages [1,5,6]. In this case report, the patient’s mother identified symptoms of urinary incontinence during the toilet training, following case series studies in which this symptom was the most prevalent [7]. The diagnostic investigation of this patient included ultrasonography of the urinary tract with dilatation of the renal pelvis and left ureter and renal scintigraphies that detected anatomical and functional abnormalities, but the diagnosis was confirmed with intraoperative retrograde pyeloureterography, with no need for more complex tests, as already mentioned in the literature [1,5]. The diagnosis occurred at four years old, earlier than described in the literature, due to the episodes of urinary tract infections and urinary incontinence, in addition to the concomitant deterioration of renal function. 

The study by Wang et al. (2022), which analyzed a series of five cases of ectopic ureters with vaginal insertion, showed a mean age at diagnosis was 31.8 years (14-46 years) and the late diagnosis was justified by the association between the rarity of the anomaly and the difficulty in identifying symptoms of urinary incontinence, confused with persistent vaginal discharge [8]. The progressive loss of kidney function can reduce urinary incontinence over time and consequently delay seeking medical care.

If there is preserved renal functionality, studies indicate a preference for nephron-sparing surgery and ureteral reimplantation [1,4]. Our patient underwent excision of the distal portion of the ureter with reimplantation in the bladder trigone. This approach was possible because, although there was a decrease in left renal function, the unit remained with a sufficient functional rate that allowed renal preservation and restored urinary continence through the surgical technique used.

## Conclusions

A single-system ectopic ureter in females is rare and the publication of case reports in the medical literature helps in the management of other patients. Diagnosis is usually delayed, sometimes made after deterioration of renal function or after late recognition of symptoms. Ectopic ureters should be included among the differential diagnosis in female patients with UTI episodes in childhood and perception of urinary incontinence during toilet training, to obtain an early diagnosis and offer the appropriate treatment.
